# The relationship between remnant cholesterol and WHO grade of pancreatic neuroendocrine neoplasms

**DOI:** 10.3389/fendo.2025.1616523

**Published:** 2025-09-04

**Authors:** Hao Zhou, Yu Wang, Yongkang Liu, Chuangen Guo, Jianhua Wang, Xiao Chen

**Affiliations:** ^1^ Department of Radiology, Affiliated Hospital of Nanjing University of Chinese Medicine, Nanjing, China; ^2^ Department of Radiology, The First Affiliated Hospital of Zhejiang University School of Medicine, Haznghozu, China

**Keywords:** pancreatic neuroendocrine tumor, cholesterol, remnant cholesterol, lipids, tumor grade

## Abstract

**Background:**

Cholesterol plays a role in tumorigenesis. However, the association between remnant cholesterol and pancreatic neuroendocrine neoplasms (PNENs) has not been clarified. In the present study, we explored the association between the remnant cholesterol level and the World Organization Health Grade of PNENs.

**Methods:**

The clinical and histopathological characteristics of PNEN patients who underwent surgery at our institution were retrospectively analyzed. Remnant cholesterol was calculated as total cholesterol - high-density lipoprotein cholesterol+low-density lipoprotein cholesterol. The remnant cholesterol/cholesterol ratio was also calculated. Grade 3 PNENs and pancreatic neuroendocrine carcinoma (PNEC) were defined as high-grade PNENs. The relationship between remnant cholesterol or the remnant cholesterol/cholesterol ratio and PNENs was analyzed using multivariable logistic regression analysis.

**Results:**

Patients with high-grade PNENs had higher remnant cholesterol levels and remnant cholesterol/cholesterol ratios than did those with low- and moderate-grade PNENs (*P* < 0.01). High proportions of patients with a remnant cholesterol concentration > 1.2 and a remnant cholesterol/cholesterol ratio > 0.26 were observed in patients with high-grade PNENs compared to those with low and moderate PNENs (27.74% vs 9.27%, *P* = 0.02; 19.56% vs 7.28%, *P* = 0.016). Multivariate logistic regression analysis revealed that remnant cholesterol was associated with high-grade PNENs (odds ratio (OR) =2.41, 95% confidence interval (CI): 1.28 - 4.56). Similar associations were observed between high-grade PNENs and a remnant cholesterol concentration greater than 1.2 (OR = 3.34, 95% CI: 1.15 - 9.68). High-grade PNENs were also associated with the remnant cholesterol/cholesterol ratio (OR = 1.45, 95% CI: 1.06 - 2.02, for continuous data; OR = 4.00, 95% CI: 1.32 - 12.09, for cholesterol/cholesterol ratio > 0.26). Similar associations were observed between the remnant cholesterol level and the remnant cholesterol/cholesterol ratio and PNEC.

**Conclusions:**

A high remnant cholesterol level and a high remnant cholesterol/cholesterol ratio were associated with high-grade PNENs or PNECs.

## Introduction

Pancreatic neuroendocrine neoplasms (PNENs) are a heterogeneous group of tumors that originate from pluripotent stem cells of the neuroendocrine system and account for approximately 2% of all pancreatic neoplasms (1). Due to advances in imaging technology, the incidence of PNENs has increased in recent years (2). Due to their heterogeneous features, the clinical manifestations, development, treatment strategies and outcomes of PNENs vary (3). The grade of PNENs is strongly associated with treatment strategies and patient prognosis (3). For low-grade tumors with a size smaller than 2 cm, observation is a preferred option if no growth occurs during surveillance. Tumors of intermediate grade may be selected for resection. For high-grade lesions that have rapidly progressive metastases, systemic therapy should be administered. Therefore, the ability of patient clinical characteristics, radiological findings and serum biomarkers, such as size, contrast enhancement characteristics and diabetes mellitus (DM), to predict high-grade PNENs has been reported (4–6).

Lipid metabolism may play important roles in cancer processes, such as stimulating cell proliferation, migration and invasion (7). Epidemiological investigations have reported a link between serum lipid levels and cancer risk (8–10). Several studies have also demonstrated that serum lipid levels are associated with more aggressive cancer (11, 12). Previous studies also showed patients with pancreatic cancer had lower serum high-density lipoprotein cholesterol (HDL-c), total cholesterol (TC) and low-density lipoprotein cholesterol (LDL-c) levels before pancreatic cancer diagnosis (13). Cholesterol, which is an essential membrane component that can produce metabolites, also plays an important role in cancer. Moreover, cholesterol-derived metabolites may affect the tumor microenvironment, support cancer progression and suppress immune responses (7). Preclinical studies have also demonstrated that blocking cholesterol synthesis and uptake affects tumor formation and growth (7, 14). Interestingly, few studies have investigated the role of cholesterol metabolism in pancreatic cancer. Xu et al. (15) reported that squalene epoxidase (SQLE), a crucial cholesterol-derived metabolite, enhanced cell proliferation, inhibited apoptosis and promoted tumor growth in pancreatic cancer. Li et al. (16) demonstrated that high levels of cholesterol esterification accumulated in human pancreatic cancer specimens and that inhibiting cholesterol esterification could suppress the growth and metastasis of pancreatic cancer. However, few studies have investigated the role of lipids in PNENs. Preclinical studies have shown the potential role of lipid lowering agents in NENs (17). Dyslipidemia has been shown to elevate cancer risk and worsen outcomes in patients with NENs (17). Several studies has examined the association between dyslipidemia and the risk of NENs (17, 18). Our previous study also reported that HDL-c was negatively associated with tumor grade and malignant behavior in PNENs (19).

It is well known that HDL-c and LDL-c predominantly transport cholesterol. Remnant cholesterol corresponds to all plasma cholesterol outside of LDL-c and HDL-c (20). In recent years, the link between remnant cholesterol and the risk or mortality of cardiovascular diseases has been widely studied (20–22). Studies have shown a positive correlation between non-HDL-c levels and the risk of high-grade PNENs (23). Remnant cholesterol contributes to but differs from non-HDL-c (20). Cholesterol may play an important role in cancer; however, the relationship between remnant cholesterol and PNENs has not been clarified. In this study, the association between remnant cholesterol and the World Health Organization (WHO) grade of PNENs was investigated.

## Materials and methods

### Patients

The study patients were described in our previous studies (19, 23). Briefly, we documented 242 PNEN patients admitted to our institution between 2011 and 2019 from the electronic medical records. After the exclusion of individuals with incomplete histological documentation or who underwent biopsy, a total of 197 patients were selected for the final analyses. This study was approved by the Institutional Review Board of the Affiliated Hospital of Nanjing University of Chinese Medicine (2017NL - 137-05). Due to its retrospective design, informed consent was not needed. Declaration of Helsinki was followed in our study.

### Clinical data collection

The following data were collected from the electronic medical records: patient demographics (age and sex), clinical features (medical history), pathological characteristics (tumor location, tumor size, mitotic count, ki67 index and tumor grade), and biochemical records (the levels of serum triglyceride (TG), TC, HDL-c, LDL-c and blood glucose). The residual cholesterol concentration was calculated as TC - HDL-c - LDL-c.

### PNEN grade

Pathological grading of PNENs was performed based on the 2019 World Health Organization classification and grading criteria. Tumor grading was based on the mitotic count and Ki-67 index. Briefly, PNENs were classified as pancreatic neuroendocrine tumor grade 1 (PNET G1): mitotic count less than 2/10 high-power field (HPF) and/or Ki-67 index less than 3%; PNET grade 2 (PNET G2): mitotic count 2 - 20/10 HPF or Ki-67 index of 3 - 20%; and grade 3 (PNEN G3) and pancreatic neuroendocrine carcinoma (PNEC): mitotic count greater than 20/10 HPF or greater than 20% Ki-67 index. PNECs included small-cell or large-cell NECs. PNEN G3 and PNECs were considered high-grade PNENs. Tumor staging was performed according to the 8th American Joint Committee on Cancer (AJCC) TNM staging criteria for pancreatic tumors.

### Statistical analysis

The commercial statistical software SPSS20 was used for the data analyses. Continuous data are presented as the mean ± standard deviation, and qualitative data are presented as numbers. Subsequently, clinicopathological variables among the patients were compared by the independent samples test or Mann–Whitney U test (continuous data), χ2 test (qualitative data), or Fisher’s exact test (qualitative data). Low and high remnant cholesterol and the remnant cholesterol/cholesterol ratio were defined by the 90th percentile of the remnant cholesterol or the remnant cholesterol/cholesterol ratio, respectively. In addition, the prevalence of high-grade PNEN was compared between patients with low and high remnant cholesterol. Univariable and multivariable logistic regression analyses were used to show the associations between remnant cholesterol levels, the remnant cholesterol/cholesterol ratio and the risk of high-grade PNEN. Remnant cholesterol and remnant cholesterol/ratio were divided into low or high group based on the 90th percentile. Restricted cubic spline regression was adopted to show the nonlinear associations. Receiver operating characteristic (ROC) curves were generated to determine the performance of the models for predicting high-grade PNENs. p values < 0.05 were considered to indicate statistical significance.

## Results

### Patient demographics and PNEN characteristics

The patient demographics and characteristics of the patients with PNENs are reported in **Table 1**. High-grade PNENs were more common in female patients and older patients (*P* < 0.01). In contrast to those with low- and moderate-grade tumors, those with high tumors were larger and had a greater Ki67 index (*P* < 0.01). The remnant cholesterol level and remnant cholesterol/cholesterol ratio were greater in patients with high-grade PNENs than in those with low- or moderate-grade PNENs (*P* < 0.01). Patients with high-grade PNENs usually had a remnant cholesterol greater than 1.2 and a remnant cholesterol/cholesterol ratio greater than 2.6 compared to those with low- and moderate-grade PNENs (*P* < 0.05). High-stage PNENs were more common than low- or moderate-grade PNENs (P < 0.05). No significant differences were detected in blood glucose, location (head/neck/body/tail), diabetes status (yes) or TG, TC, HDL-c or LDL-c levels (*P* > 0.05).

**Table 1:** Characteristics of patients with pancreatic neuroendocrine neoplasms.

	High grade (n = 46)	Low and moderate grade (n = 151)	*P*
Age (years)	60.41± 10.06	54.73 ± 11.81	0.004
Sex (male/female)	13/33	80/71	0.003
Tumor size (cm)	4.49 ± 2.93	3.04 ± 1.92	<0.001
Ki67 index	52.86 ± 23014	4.29 ± 4.32	<0.001
TG (mmol/L)	1.42 ± 0.80	1.36 ± 0.86	0.75
TC (mmol/L)	4.49 ± 1.14	4.30 ± 1.03	0.27
HDL-c (mmol/L)	1.09 ± 0.41	1.16 ± 0.35	0.22
LDL-c (mmol/L)	2.42 ± 0.87	2.45 ± 0.81	0.82
Remnant cholesterol (mmol/L)	0.98 ± 1.02	0.68 ± 0.40	0.003
Remnant cholesterol > 1.2	10	14	0.02
Remnant cholesterol/cholesterol ratio	0.21 ± 0.18	0.16 ± 0.07	0.004
Remnant cholesterol/ cholesterol > 0.26	9	11	0.016
Blood glucose (mmol/L)	5.69 ± 1.24	5.49 ± 1.98	0.52
Location (head-neck/body/tail)	24/13/9	69/53/29	0.67
Diabetes (yes)	12	30	0.16
Tumor stage			
T1/T2/T3/T4	3/25/18/0	59/59/33/0	<0.001
N0/N1/N2	36/10/0	143/8/0	0.001
M0/M1	46/0	151/0	/

HDL-c, high-density lipoprotein cholesterol; PNECs, pancreatic neuroendocrine carcinomas; LDL-c, low-density lipoprotein cholesterol; PNENs, pancreatic neuroendocrine neoplasms; TC, total cholesterol; TG, triglyceride.

**Figure 1:**
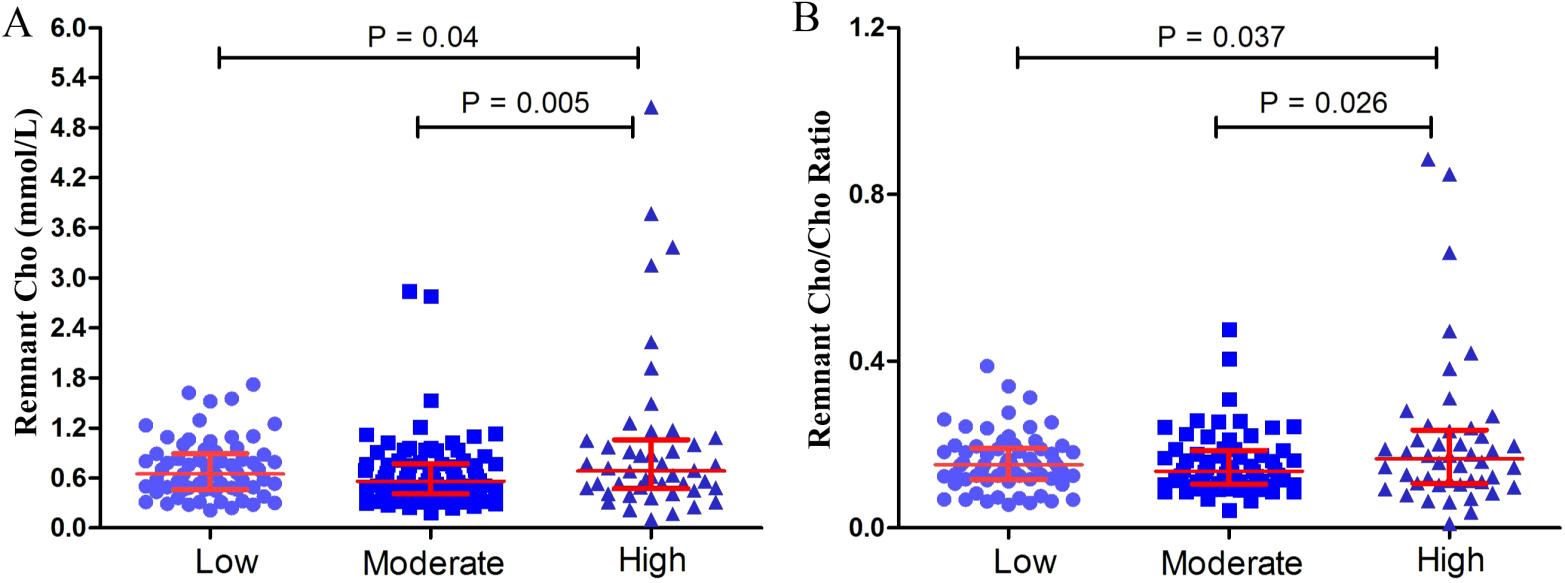
Remnant cholesterol (Cho) **(A)** and the remnant Cho/Cho ratio **(B)** in low-grade (grade 1, n = 66), moderate-grade (grade 2, n = 85) and high-grade (grade 3 and pancreatic neuroendocrine carcinomas, n = 46) PNENs.

The remnant cholesterol level and remnant cholesterol/cholesterol ratio in low-, moderate- and high-grade PNENs are shown in **Figure 1**. The remnant cholesterol and the remnant cholesterol/cholesterol ratio were greater in high-grade PNENs (Grade 3 and PNEC) than in low-grade PNENs (Grade 1) and moderate-grade PNENs (Grade 2) (*P* < 0.05).

### Association between remnant cholesterol and high-grade PNENs

**Figure 2:**
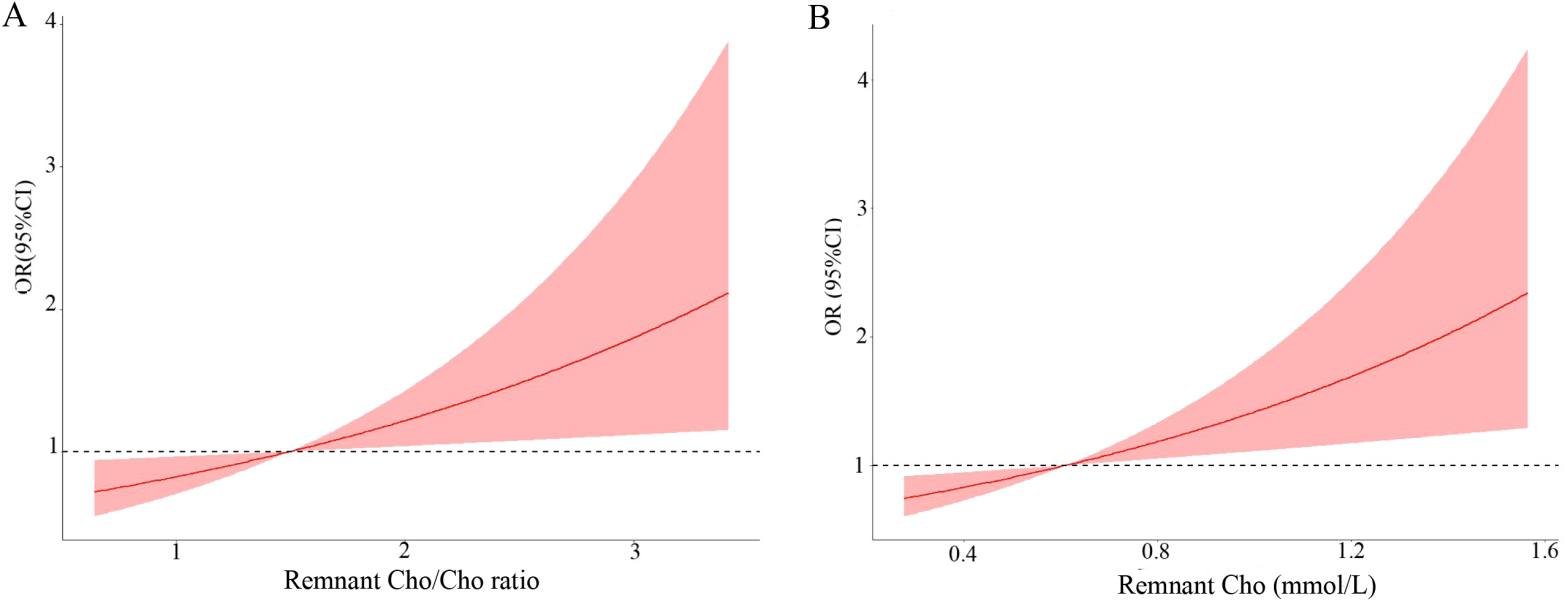
Multivariable adjusted odds ratio for high-grade PNENs according to the level of remnant cholesterol (Cho) **(A)** and the remnant cholesterol/cholesterol ratio **(B)**. Age, sex, lymph node metastasis status and tumor size were adjusted (n = 197).

The associations between remnant cholesterol and high-grade PNENs are shown in **Table 2**. Tumor size, patient age, sex and remnant cholesterol (odds ratio (OR) = 2.26, 95% confidence interval (CI): 1.23 - 4.21) were associated with high-grade PNENs. After further adjustment for lymph node metastasis and DM, remnant cholesterol was still independently associated with high-grade PNENs (OR = 2.41, 95% CI: 1.28 - 4.56). A remnant cholesterol concentration > 1.2 mmol/L was also associated with high-grade PNENs (OR = 3.34, 95% CI: 1.15 - 9.68). The risk of high-grade PNENs also increased with increasing remnant cholesterol levels according to restricted cubic spline analysis (**Figure 2A**).

**Table 2:** The association between the remnant cholesterol level and high-grade PNENs.

	Variables	Model 1	*P*	Model 2	*P*
		OR (95%CI)		OR (95%CI)	
Continuous data	Tumor size (cm)	1.46 (1.21 - 1.75)	<0.001	1.39 (1.15 - 1.68)	0.001
	Age (years)	1.05 (1.02 - 1.09)	0.005	1.05 (1.01 - 1.09)	0.007
	Sex (female vs male)	0.27 (0.12 - 0.62)	0.002	0.25 (0.11 - 0.59)	0.002
	Remnant cholesterol (mmol/L)	2.26 (1.23 - 4.21)	0.009	2.41 (1.28 - 4.56)	0.007
Categorical data	Tumor size (cm)	1.38 (1.15 - 1.67)	0.001	1.34 (1.10 - 1.62)	0.003
	Age (years)	1.06 (1.02 - 1.10)	0.002	1.06 (1.02 - 1.10)	0.002
	Sex (female vs male)	0.28 (0.13 - 0.63)	0.002	0.25 (0.11 - 0.59)	0.002
	Remnant cholesterol > 1.2 mmol/L	3.25 (1.16 - 9.10)	0.025	3.34 (1.15 - 9.68)	0.026

Model 2 multivariable analysis further adjusted for lymph node metastasis and diabetes status.

CI, confidence interval; OR, odds ratio.

**Table 3** further shows the link between remnant cholesterol or remnant cholesterol/cholesterol and PNEC. Multivariate regression analyses demonstrated that remnant cholesterol levels were independently associated with PNEC (OR = 2.20, 95% CI: 1.13 - 4.26; OR = 1.41, 95%CI:1.01-1.99). Interestingly, remnant cholesterol levels or remnant cholesterol/cholesterol ratio were also independently associated with well-differentiated PNEN G3 (OR = 2.86, 95% CI: 1.40 - 5.83; OR = 1.51, 95%CI: 1.08 - 2.11) (**Table 4**).

**Table 3:** The association between remnant cholesterol or the remnant cholesterol/cholesterol ratio and PNEC.

	Variables	Model 1	*P*	Model 2	*P*
		OR (95%CI)		OR (95%CI)	
Remnant cholesterol	Tumor size (cm)	1.20 (0.99 - 1.45)	0.06	1.16 (0.96 - 1.40)	0.12
	Age (years)	1.06 (1.01 - 1.11)	0.02	1.06 (1.01 - 1.12)	0.02
	Sex (female vs male)	0.29 (0.09 - 0.88)	0.03	0.19 (0.05 - 0.70)	0.01
	Remnant cholesterol	2.15 (1.14 - 4.05)	0.017	2.20 (1.13 - 4.26)	0.020
Remnant cholesterol/ cholesterol ratio	Tumor size (cm)	1.19 (0.99 - 1.43)	<0.001	1.15 (0.96 - 1.39)	0.13
	Age (years)	1.06 (1.01 - 1.12)	0.002	1.07 (1.01 - 1.13)	0.015
	Sex (female vs male)	0.29 (0.10 - 0.88)	0.002	0.19 (0.05 - 0.68)	0.011
	Remnant cholesterol/cholesterol ratio (×10)	3.65 (1.00 - 1.95)	0.048	1.41 (1.01 - 1.99)	0.047

Model 2 was further adjusted for lymph node metastasis and diabetes.

CI, confidence interval; OR, odds ratio.

**Table 4:** The association between remnant cholesterol or the remnant cholesterol/cholesterol ratio and well-differentiated G3 PNENs.

	Variables	Model 1	*P*	Model 2	*P*
		OR (95%CI)		OR (95%CI)	
Remnant cholesterol	Tumor size (cm)	1.46 (1.21 - 1.75)	< 0.001	1.42 (1.17 - 1.71)	0.009
	Age (years)	1.05 (1.02 - 1.09)	0.005	1.05 (1.01 - 1.09)	0.02
	Sex (female vs male)	0.27 (0.12 - 0.62)	0.002	0.22 (0.09 - 0.54)	0.001
	Remnant cholesterol	2.28 (1.23 - 4.21)	0.009	2.86 (1.40 - 5.83)	0.004
Remnant cholesterol/ cholesterol ratio	Tumor size (cm)	1.44 (1.20 - 1.73)	< 0.001	1.39 (1.16 - 1.68)	0.001
	Age (years)	1.05 (1.02 - 1.09)	0.004	1.05 (1.01 - 1.11)	0.014
	Sex (female vs male)	0.27 (0.12 - 0.61)	0.002	0.23 (0.10 - 0.54)	0.001
	Remnant cholesterol/cholesterol ratio (×10)	1.43 (1.04 - 1.96)	0.027	1.51 (1.08 - 2.11)	0.015

Model 2 was further adjusted for lymph node metastasis and diabetes.

CI, confidence interval; OR, odds ratio.

### High-grade PNENs are associated with a high cholesterol/cholesterol ratio

Subsequently, we reported a link between the remnant cholesterol/cholesterol ratio and the risk of high-grade PNENs (**Table 5**). A high cholesterol/cholesterol ratio (×10) was independently associated with high-grade PNENs (OR = 1.43, 95% CI: 1.04 – 1.96). Similar results were observed after further adjusting for lymph node metastasis and DM (OR = 1.45, 95% CI: 1.06 - 2.02). A high cholesterol/cholesterol ratio (greater than 2.6) was also associated with high-grade PNENs (OR = 4.00, 95% CI: 1.32 - 12.09). The risk of high-grade PNENs also increased with the cholesterol/cholesterol ratio according to restricted cubic spline analysis (**Figure 2B**). **Table 3** further shows a link between the remnant cholesterol/cholesterol ratio and PNEC. Multivariate regression analyses demonstrated that the remnant cholesterol/cholesterol ratio was associated with PNEC (OR = 1.41, 95% CI: 1.01 - 1.99).

**Table 5:** The association between the remnant cholesterol/cholesterol ratio and high-grade PNENs.

	Variables	Model 1	*P*	Model 2	*P*
		OR (95%CI)		OR (95%CI)	
Continuous data	Tumor size (cm)	1.44 (1.20 - 1.73)	<0.001	1.38 (1.15 - 1.67)	0.001
	Age (years)	1.05 (1.02 - 1.09)	0.004	1.05 (1.01 - 1.09)	0.006
	Sex (female vs male)	0.27 (0.12 - 0.61)	0.002	0.25 (0.10 - 0.58)	0.001
	Remnant cholesterol/chXolesterol ratio (×10)	1.43 (1.04 - 1.96)	0.027	1.45 (1.06 - 2.02)	0.019
Categorical data	Tumor size (cm)	1.43 (1.19 - 1.72)	<0.001	1.36 (1.13 - 1.65)	0.001
	Age (years)	1.06 (1.02 - 1.10)	0.002	1.06 (1.02 - 1.10)	0.002
	Sex (female vs male)	0.29 (0.13 - 0.63)	0.002	0.26 (0.11 - 0.60)	0.002
	Remnant cholesterol/cholesterol ratio > 0.26	3.65 (1.23 - 10.90)	0.020	4.00 (1.32 - 12.09)	0.014

Model 2 was further adjusted for lymph node metastasis and diabetes.

CI, confidence interval; OR, odds ratio.

### Models for predicting high-grade PNENs

**Figure 3:**
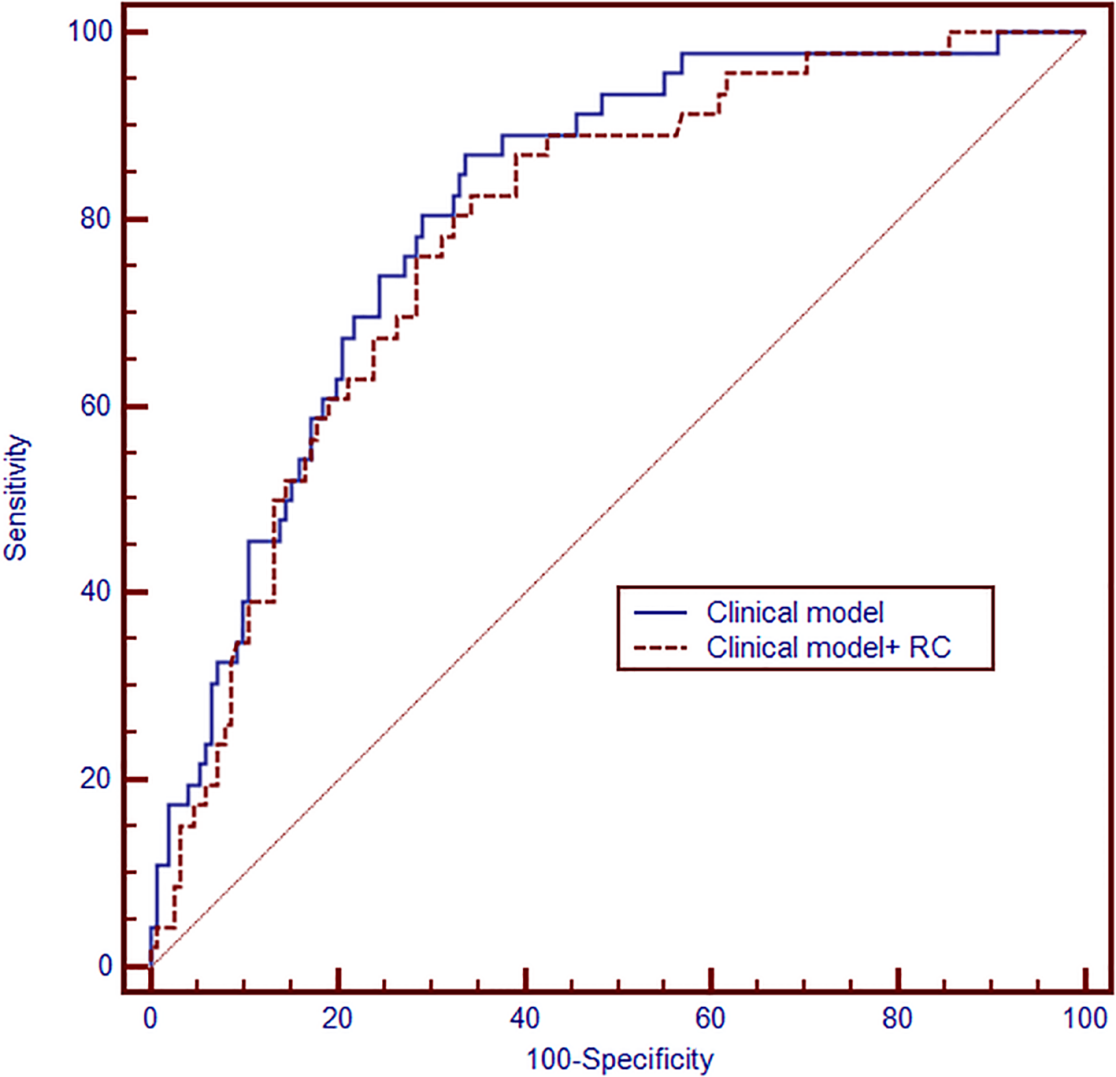
Receiver operating characteristic (ROC) curve showing the performance of the models for predicting high-grade PNENs (n = 197). The clinical model was based on tumor size, sex and age (area under the curve(AUC) = 0.78, 95% CI: 0.72 - 0.84). Then, the remaining cholesterol was added to develop another model (AUC = 0.81, 95%CI:0.74-0.87).

Subsequently, we established two models for the prediction of high-grade PNENs using receiver operating characteristic (ROC) curves based on the independently associated factors obtained from logistic regression analyses (**Figure 3**). The model based on tumor size, patient age, and sex had an area under the curve (AUC) of 0.78 (95% CI: 0.72 - 0.84). The addition of remnant cholesterol improved the AUC to 0.81 (95% CI: 0.74 - 0.87).

## Discussion

Cholesterol and cholesterol-derived metabolites are important in tumorigenesis and progression (24). However, the link between remnant cholesterol and pancreatic tumors has not been well studied. Our results showed that the remnant cholesterol level and the remnant cholesterol/cholesterol ratio were positively associated with high-grade PNENs. The residual cholesterol-based model also had good performance in predicting high-grade PNENs. Our study revealed novel factors associated with high-grade PNENs or PNECs, and the remnant cholesterol may be valuable for PNEN management, such as early screening, effective treatment strategy and putative molecular targets.

Epidemiological studies have shown that serum cholesterol levels are associated with certain cancer risks (25). However, our study did not observe a significant association between TC and the risk of high-grade PNENs or PNECs (data not shown). Our previous studies reported associations between high-density lipoprotein cholesterol (HDL-c) and non-HDL-c and malignancy or risk of high-grade PNENs (19, 23). Interestingly, increasing amounts of data indicate that remnant cholesterol is causally associated with atherosclerosis and cardiovascular mortality (22, 26). The level of remnant cholesterol is obviously different from that of non-HDL-c. However, the associations between remnant cholesterol, the cholesterol content of TG-rich lipoproteins, and cancer are not well understood. Recent studies have demonstrated that elevated remnant cholesterol levels contribute to the mortality of certain types of cancer (27, 28). Some studies showed that the lipid content in pancreatic cancer was higher than that in chronic pancreatitis (29) which supported that high lipid may be associated malignant lesions. However, to the best of our knowledge, our study may be the first to show an association between remnant cholesterol and pancreatic tumors.

Clinical models have been reported to identify high grade PNENs (30, 31), including imaging models and radiomics models. Our study demonstrated that clinical models based on age, tumor size and sex also have potential value. More importantly, our data indicate that adding remnant cholesterol markedly improved the routine clinical model’s performance (AUC rose from 0.78 to 0.81), approaching the accuracy reported for imaging models (32). Nevertheless, this improvement must be interpreted cautiously, given the small sample size and the risk of overfitting inherent to the limited dataset.

How cholesterol affects cancer has been widely investigated. Cholesterol is thought to be necessary for cancer cell proliferation and survival (24). An increase in intracellular cholesterol levels is associated with an increase in cancer aggressiveness (33). Increasing mitochondrial cholesterol levels lead to resistance to apoptosis in cancer cells (34). Cholesterol may affect tumor cells by regulating immune responses and tumor cell stemness, inducing ferroptosis and autophagy, and attenuating the DNA repair process (24). There is also an association between cholesterol metabolites and the risk of developing various types of cancer (24, 35). The steroids and oxysterols synthesized from cholesterol play important roles in cancer development or have anti-proliferative effects on cancer cells (25). Some studies have also shown the role of cholesterol-related factors in pancreatic tumors (36). Acetyl coenzyme A is a key molecule involved in cholesterol biosynthesis and promotes the formation of acinar-to-ductal metaplasia (ADM) and the development of pancreatic intraepithelial neoplasia (PanIN) (37). SQLE, a crucial cholesterol-derived metabolite, promotes pancreatic cancer cell proliferation and cancer development (15, 38). In addition, studies reported aberrant lipoprotein receptor expression in multiple cancers, including gastrointestinal malignancies like liver and pancreatic tumors (17). Those receptor also associated with signaling pathways that regulate both cancer cells and their tumor microenvironment (17). Furthermore, circulating remnant cholesterol concentrations demonstrate a significant association with systemic inflammatory markers (39), which is a risk factor of cancers (40). Chronic inflammation can cause cancer by many pathway, such as nuclear factor-κB and STAT3 signaling (41). Abrogating cholesterol esterification can inhibit pancreatic cancer cell growth (16). However, the exact mechanisms by which remnant cholesterol affects cancer are incompletely understood. These cholesterol-related pathways may also be involved in the development of high-grade PNENs.

## Strengths and limitations of the study

Our study had limitations. First, our study only reported the associations. The underlying cellular or molecular mechanisms were not studied because there were no commercial remnant cholesterol. Second, the sample size was relatively small because PNENs are rare types of pancreatic tumors and most of cases were selected from single center. Selection bias cannot be avoided. The generalizability of our findings require further validation. Our results should be confirmed in a study with a larger sample size. Third, although we controlled for several variables, residual confounding effects from other factors, such as dietary habits and body mass index, cannot be ruled out. In addition, we did not perform an external validation because of the low prevalence of PNENs. The reproducibility of our findings require further validation. Finally, survival data and recurrence were not obtained in our patients, therefore we did not observe an association between the remnant cholesterol or the remnant cholesterol/cholesterol ratio and the outcomes of PNENs.

## Conclusion

In summary, our research showed that the remnant cholesterol level and the remnant cholesterol/cholesterol ratio were independently associated with high-grade PNENs. The residual cholesterol or the remnant cholesterol/cholesterol ratio may serve as risk factors or associated factors for managing high-grade PNENs. However, further research is needed to elucidate the mechanisms that explain the association between remnant cholesterol and malignant pancreatic tumors.

## Data Availability

The raw data supporting the conclusions of this article will be made available by the authors, without undue reservation.
