# Supplementary figures and images for: The relationship between remnant cholesterol and WHO grade of pancreatic neuroendocrine neoplasms
Source: Front Endocrinol (Lausanne). 2025 Sep 4;16:1616523. doi: 10.3389/fendo.2025.1616523 (PMC12443567; doi:10.3389/fendo.2025.1616523)

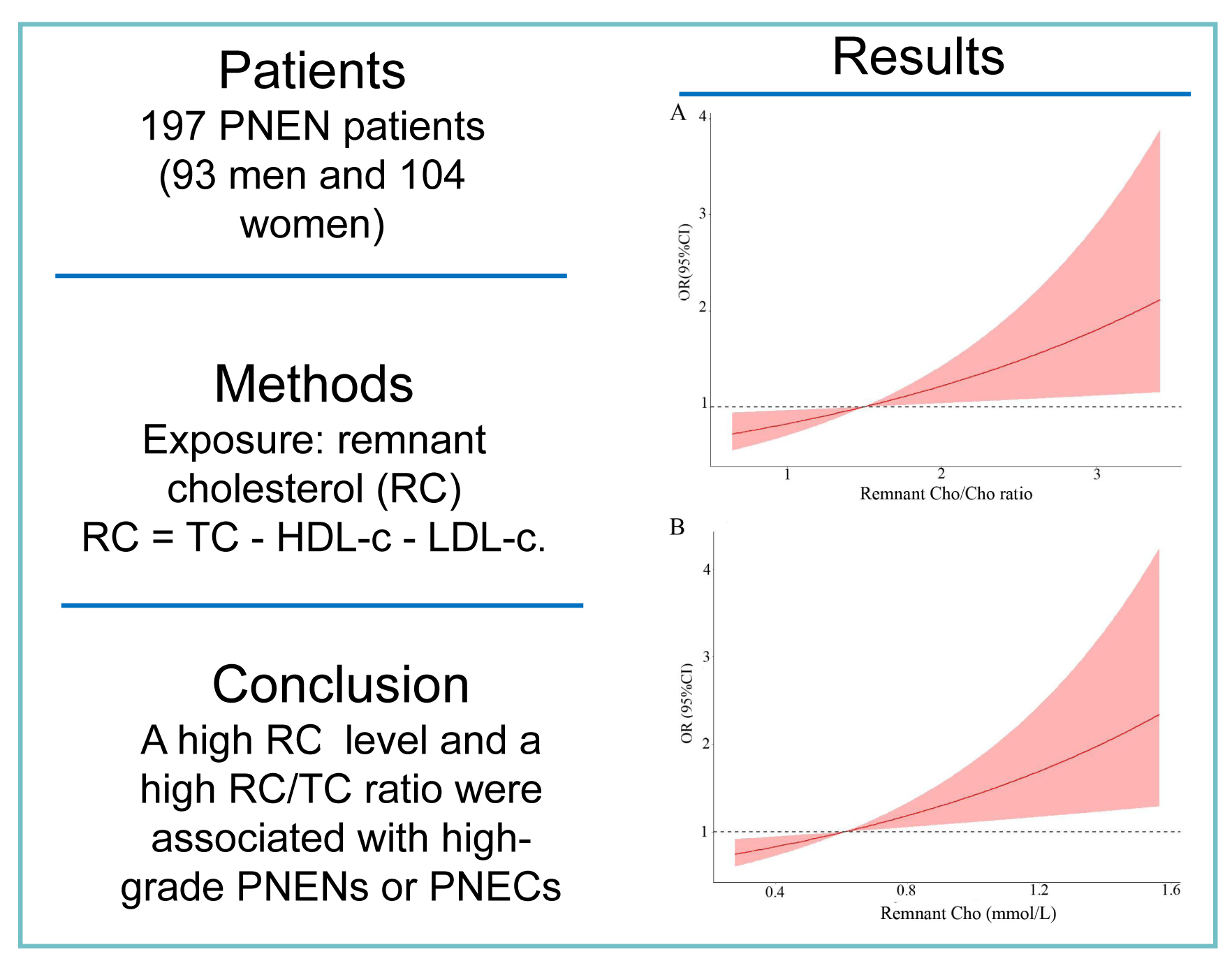

Supplement: Supplementary file 1 [file Image1.tif]
